# Supplementary material for: WordCommentsAnalyzer: A windows software tool for qualitative research
Source: F1000Res. 2018 Sep 4;7:536. Originally published 2018 May 3. [Version 2] doi: 10.12688/f1000research.14819.2 (PMC6137411; doi:10.12688/f1000research.14819.2)
Supplement: Supplementary file 3 [file f1000research-7-17614-s0002.tgz › b2a79496-4ada-47c4-9682-a99ce867836d.zip!/Eight Iranian Health Journals/Salmand_Iranian Journal of Ageing _37.docx]

© Salmand_Iranian Journal of Ageing . This work is licensed under CC BY-NC 4.0 (https://creativecommons.org/licenses/by-nc/4.0/).

Volume & Issue: دوره 4 - شماره 2

Title: کیفیت زندگی و ویژگی‌ های جمعیت‌ شناختی مرتبط با آن در سالمندان شهر تهران

http://salmandj.uswr.ac.ir/article-1-327-fa.html

اهداف: هدف از پژوهش حاضر بررسی کیفیت زندگی و خصوصیات جمعیت شناختی مرتبط با آن در جمعیت سالمندان شهر تهران بود. مواد و روش‌ها: این پژوهش از نوع توصیفی-همبستگی بود. در این مطالعه 243 سالمند با روش نمونه گیری در دسترس از مراجعان به کانون های سالمندان فرهنگسراها و پارک های مناطق مختلف تهران در سال 86 انتخاب شدند. ابزارهای مورد استفاده عبارت بودند از پرسشنامه «خصوصیات جمعیت شناختی» و «پرسشنامه کیفیت زندگی WHOQoL-BREF». جهت تجزیه و تحلیل داده ها از آمار توصیفی و رگرسیون گام به گام استفاده شد. یافته‌ها: در این تحقیق 134 مرد و 109 زن سالمند شرکت داشتند که میانگین سنی آنها 71/41 بود. نتایج تحلیل رگرسیون نشان داد که در حوزه سلامت جمعی، روانی و روابط اجتماعی، روابط خانوادگی خوب پیش بینی کننده مثبت و به ترتیب بیماری جسمی، روانی و روابط خانوادگی ضعیف پیش بینی کننده کاهش نمرات بود. در حوزه محیط، سطح اقتصادی خوب، پیش بینی کننده افزایش نمرات و روابط خانوادگی ضعیف، پیش بینی کننده کاهش نمرات بود. نتیجه‌گیری: بر طبق یافته ها مشخص شد که روابط خانوادگی یکی از مهمترین عوامل تاثیرگذار بر حوزه های مختلف کیفیت زندگی است.

Title: تغییرات پارامترهای فضایی-زمانی در راه رفتن مردان سالمند

http://salmandj.uswr.ac.ir/article-1-325-fa.html

اهداف: در مطالعه حاضر برخی از پارامترهای فضایی – زمانی راه رفتن مردان مسن و مقایسه آن با مردان جوان مورد بررسی قرار گرفت. مواد و روش‌ها: 57 مرد سالمند سالم (میانگین سنی 72±5/5 سال، قد 170±0/6 متر و وزن 75±10/3 کیلوگرم) و 57 مرد جوان سالم (میانگین سنی 25±8/5 سال، قد 1.75±0/5 متر و وزن 78±13/1 کیلوگرم) در این مطالعه شرکت کردند. مدل چهار قسمتی متشکل از تنه، ران، ساق و پا با استفاده از ده نشانگر رفلکسی برای معرفی اندام های آزمودنی ها مورد استفاده قرار گرفت. اطلاعات کینماتیکی با استفاده از چهار دوربین با سرعت بالا (Hz90) جمع آوری گردید. برای تجزیه و تحلیل داده ها از نرم افزار SPSS و آزمون t مستقل (α≤0/05) استفاده شد. یافته‌ها: نتایج نشان داد که سرعت (28%)، آهنگ گام برداری (13%)، طول گام (15%) در افراد مسن به طور معنی داری کمتر و فاز استقرار (2%) و زمان آغاز حرکت به جلو (4%) به طور معنی داری بیشتر از افراد جوان است در حالی که در زمان حمایت دوگانه دو گروه تفاوت معنی داری مشاهده نشد. نتیجه‌گیری: تفاوت های مشاهده شده را می توان به کاهش انعطاف پذیری عضلات، دامنه حرکتی مفاصل و کنترل عصبی عضلانی که در خصوص سالمندان گزارش شده است مرتبط دانست.

Title: ارتباط سندرم متابولیک، پروتئین واکنشی C و بیماری مزمن کلیوی در سالمندان آسایشگاه کهریزک

http://salmandj.uswr.ac.ir/article-1-324-fa.html

اهداف: در این مطالعه ارتباط بینابینی میان پروتئین واکنشی C، سندرم متابولیک و بیماری مزمن کلیوی در گروهی از سالمندان ایرانی مورد بررسی قرار گرفت. مواد و روش‌ها: در این مطالعه مقطعی، 122 سالمند از میان 1220 سالمند مقیم آسایشگاه خیریه کهریزک با میانگین سنی 73/6±9/71 مورد مطالعه قرار گرفتند. سندرم متابولیک و بیماری مزمن کلیوی به ترتیب بر اساس شاخص های برنامه آموزش ملی کلسترول – پانل درمانی بزرگسالان III و میزان فیلتراسیون گلومرولی تخمین زده شده 60< میلی متر در دقیقه در 1/73 متر مربع تعریف شدند. پروتئین واکنشی C³3 میلی گرم در دسی لیتر بعنوان پروتئین واکنشی C بالا در نظر گرفته شد. یافته‌ها: بیماری مزمن کلیوی در نمونه های دارای سندرم متابولیک و بدون سندرم متابولیک به ترتیب 82/9 و 59/3 درصد بود (P<0/006). نمونه های دارای سندرم متابولیک و پروتئین واکنشی C بالا نسبت به نمونه های بدون سندرم متابولیک و پروتئین واکنشی C پایین، از شانس 1/71 برابری بیشتری برای داشتن بیماری مزمن کلیوی برخوردار بودند. نتیجه‌گیری: سندرم متابولیک عامل خطری برای بیماری مزمن کلیوی در جمعیت مسن این مطالعه بود. همچنین نسبتهای شانس بیماری مزمن کلیوی در حضور پروتئین واکنشی C بالا و سندرم متابولیک، به طور معنی داری افزایش یافت.

Title: اضافه وزن به‌عنوان عامل محافظتی در برابر وقوع زوال عقل در سالمندان 80 سال و بالاتر آسایشگاه کهریزک

http://salmandj.uswr.ac.ir/article-1-323-fa.html

اهداف: در این مطالعه، ارتباط نمایه توده بدنی و دور کمر در سنین سالمندی با خطر وقوع زوال عقل در گروهی از سالمندان ایرانی بررسی شد. مواد و روش‌ها: در مطالعه مقطعی حاضر 107 فرد مسن 80 سال مقیم بنیاد خیریه کهریزک مورد بررسی قرار گرفتند. برای ارزیابی زوال عقل از پرسشنامه «بررسی مختصر وضعیت شناختی» استفاده شد و نمایه توده بدنی به صورت وزن (کیلوگرم) تقسیم بر مجذور قد (سانتی متر) محاسبه شد. یافته‌ها: در یک مدل رگرسیون لوجستیک چند متغیری، اضافه وزن در سنین سالمندی به عنوان یک عامل محافظتی مستقل و معنی دار زوال عقل معرفی شد (نسبت شانس 0.24، فاصله اطمینان %95، 0.06-0/93)، همچنین هیچ رابطه معنی داری بین دور کمر و خطر وقوع زوال عقل وجود نداشت. نتیجه‌گیری: اضافه وزن در سالمندی به عنوان یک عامل محافظتی در برابر خطر وقوع زوال عقل نشان داده شد.

Title: اثر روغن ماهی بر سطح سرمی فراسنج‌های لیپیدی، لیپوپروتئین a، آپولیپوپروتئین A-1 و B، قندخون و انسولین ناشتا، و مقاومت به انسولین در سالمندان آسایشگاه کهریزک

http://salmandj.uswr.ac.ir/article-1-322-fa.html

اهداف: این مطالعه به منظور بررسی اثر اسیدهای چرب امگا-سه بر فراسنج های لیپیدی، لیپوپروتئین (a)، آپولیپوپروتئین های A-1 و B، قند خون، انسولین ناشتا و مقاومت به انسولین در گروهی از سالمندان ایرانی صورت گرفت. مواد و روش‌ها: در این کارآزمایی بالینی 124 سالمند 65 سال و بالاتر ساکن آسایشگاه خیریه کهریزک به طور تصادفی به 2 گروه مداخله (با مصرف روزانه 1 گرم کپسول روغن ماهی) و کنترل (با مصرف دارونما) تقسیم شدند. در آغاز و پایان مطالعه از همه آزمودنی ها، پیش آزمون و پس آزمون از طریق خون گیری و فرسنجهای لیپیدی به عمل آمد. یافته‌ها: در گروه کنترل در پایان ماه ششم، سطح تری گلیسرید خون و آپو A-1 به طور معنی داری افزایش (به ترتیب P=0/01 و P=0/02) و سطح کلسترول لیپوپروتئین با دانسیته بالا به طور معنی داری کاهش یافت (P=0/009). اثرات کلی و مقایسه ای روغن ماهی در برابر دارونما به طور مثبتی معنی دار بود (P=0/02). نتیجه‌گیری: دریافت روغن ماهی در سالمندان در کاهش تری گلیسرید می تواند موثر باشد.

Title: تأثیر یک دوره تمرین منتخب هوازی بر سطوح هورمون‌های استروژن، پاراتیروئید، کلسیم، آلکالن فسفاتاز و آلبومین سرم زنان سالمند

http://salmandj.uswr.ac.ir/article-1-321-fa.html

اهداف: این تحقیق با هدف بررسی تاثیر هشت هفته تمرین منتخب هوازی بر هورمون استروژن، پاراتیروئید، کلسیم، آلکالن فسفاتاز و آلبومین سرم زنان سالمند انجام گرفت. مواد و روش‌ها: تعداد 22 نفر از زنان یائسه (70-55 سال) عضو کانون جهاندیدگان شهر تهران انتخاب و سپس به طور تصادفی در دو گروه تمرین هوازی (12 نفر) و کنترل (10 نفر) قرارگرفتند. برنامه تمرین هوازی شامل 8 هفته انجام حرکات جاگینگو حرکات ایروبیکی با شدت 60-70 درصد حداکثر ضربان قلب، سه روز در هفته بود. خون گیری در دو مرحله، 24 ساعت قبل از شروع اولین جلسه تمرین (پیش آزمون) و 24 ساعت پس از آخرین جلسه تمرین (پایان هفته هشتم) انجام گرفت. برای تعیین میزان کلسیم سرم (Ca) از روش رنگ سنجی شیمیایی (کرزول فتالئین)، هورمون پاراتیروئید و استروژن از روش ELISA، آلکالن فسفاتاز از روش رنگ سنجی سینتیکی با استفاده از پارانیترو فسفات و برای تعیین آلبومین سرم هر نمونه خونی از روش رنگ سنجی شیمیایی (بروم کرزول گرین) استفاده گردید. همچنین جهت تجزیه و تحلیل داده ها از آزمون آماری t همبسته و مستقل استفاده شد. یافته‌ها: نتایج تحقیق، افزایش معنی داری را در میزان هورمون پاراتیروئید و آلکالن فسفاتاز سرمی، پس از تمرین نشان داد (P≤0/05). از سوی دیگر میزان کلسیم تام و آلبومین پلاسما کاهش یافت اما این اختلاف معنی دار نبود (P≥0/05). همچنین میزان استروژن پس از تمرین کاهش یافت اما این کاهش معنی دار نبود (P≥0/05). نتیجه‌گیری: با توجه به نتایج حاصل از این پژوهش، به نظر می رسد هشت هفته تمرین منتخب هوازی بر نشانگرهای ساخت استخوان تاثیر بگذارد. به هر حال این نتایج ممکن است نشان دهنده اثرات آنابولیک تمرین هوازی بر ساخت استخوان در زنان سالمند باشد. نتایج این تحقیق از اثرات مثبت تمرین بر افزایش توده استخوانی حمایت می کند.

Title: ارتباط وضعیت شناختی و توانایی عملکردی در سالمندان آسایشگاه خیریه کهریزک

http://salmandj.uswr.ac.ir/article-1-317-fa.html

اهداف: در مطالعه حاضر، ارتباط بین اختلال شناختی و اجزای عملکرد روزانه در سالمندان ساکن آسایشگاه خیریه کهریزک مورد مطالعه قرار گرفت. مواد و روش‌ها: در یک مطالعه مقطعی با شرکت 184 نفر از سالمندان آسایشگاه کهریزک وضعیت شناختی و عملکرد روزانه آنها به ترتیب با استفاده از پرسشنامه های «بررسی مختصر وضعیت شناختی» و «بارتل» مورد ارزیابی قرار گرفت. یافته‌ها: داده ها نشان داد 31/7% از شرکت کنندگان وضعیت شناختی طبیعی و 63/3%، دچار اختلال شناختی بودند. ارتباط بین وضعیت شناختی و وضعیت عملکرد روزانه بعد از تصحیح عوامل مداخله گر معنی دار بود (r=0/2 و P=0/01). با استفاده از رگرسیون لجستیک چند گانه، ارتباط بین اختلال شناختی و اختلال عملکردی سنجیده شد (P=0/05). نسبت شانس برای اختلال عملکردی روند رو به تزایدی با افزایش شدت اختلال شناختی داشت (برای اختلال شناختی خفیف =1/13 نسبت شانس با فاصله اطمینان 0/54-2/37 و برای اختلال شناختی متوسط تا شدید =3/67 نسبت شانس با فاصله اطمینان 0/39-34/35). ارتباط بین اختلال اجزای بارتل و وضعیت شناختی نشان داد بین توانایی استحمام و وضعیت شناختی (=2/33 نسبت شانس با فاصله اطمینان 1/14-4/72) و همچنین بین توانایی حرکت کردن و وضعیت شناختی (=2/96 نسبت شانس با فاصله اطمینان 1/30-6/75)، معنی دار بود. نتیجه‌گیری: به نظر می رسد وضعیت شناختی می تواند عامل پیش بینی کننده وضعیت عملکرد روزانه در سالمندان باشد.

Title: شناسایی سالمندان درمعرض خطر زمین خوردن، به وسیله آزمون های تعادل تحت شرایط تکالیف دوگانه

http://salmandj.uswr.ac.ir/article-1-316-fa.html

اهداف: پژوهش حاضر با هدف بررسی کفایت آزمون تعادل تحت شرایط تکالیف دوگانه برای شناسایی سالمندان با و بدون سابقه زمین خوردن انجام شد. مواد و روش‌ها: تحقیق حاضر از نوع علی-مقایسه ای بود. بدین منظور تعداد 41 سالمند مرد از سه پارک شهر تهران بعد از تکمیل پرسشنامه ارزیابی هشیاری به صورت در دسترس انتخاب شدند. آزمودنی ها شامل 20 سالمند بدون سابقه زمین خوردن (75/95±6/28 سال) و 21 سالمند با سابقه زمین خوردن (72/50±7/31 سال) بودند. همه آزمودنی ها آزمون برخاستن و حرکت کرن زماندار، تحت سه شرایط تکالیف مجرد (خود آزمون)، تکالیف دوگانه شناختی-تعادل (اجرای آزمون همراه با شمارش معکوس اعداد تصادفی) و تکلیف دوگانه حرکتی-تعادل (اجرای آزمون همراه با حمل فنجان پر آب) انجام دادند. تجزیه و تحلیل داده ها با استفاده از تحلیل واریانس چند متغیری و رگرسیون لوجستیک در سطح معنی داری P<0/05 انجام شد. یافته‌ها: نتایج نشان داد که تفاوت معناداری بین گروه های سالمند با و بدون سابقه زمین خوردن در متغیر وابسته ترکیبی خطر زمین خوردن وجود داشت (P=0/0005) به طوری که سالمندان بدون سابقه زمین خوردن در همه آزمونها وضعیت بهتری داشتند. همچنین نشان داده شد که تکلیف دوگانه شناختی-تعادل قابلیت پیش بینی زمین خوردن سالمندان را داشت (P=0/013). نتیجه‌گیری: به طور کلی می توان آزمون برخاستن و حرکت کردن زماندار تحت شرایط تکلیف دوگانه شناختی را به عنوان روش مناسبی برای شناسایی خطر زمین خوردن سالمندان به کار برد.
